# The complete chloroplast genome sequence of *Senna tora* and phylogenetic analysis

**DOI:** 10.1080/23802359.2020.1781573

**Published:** 2020-09-29

**Authors:** Qing Xu, Lirong Ma, Guo Chen

**Affiliations:** a College of Life Sciences, Northwest University, Xi’an, China; b Department of Biopharmaceutics, School of Pharmacy, The Fourth Military Medical University, Xi’an, China

**Keywords:** *S. tora*, chloroplast genome, phylogenetic analysis, genetic information

## Abstract

The complete chloroplast genome sequence of *Senna tora* was characterized from Illumina pair-end sequencing. The chloroplast genome of *S. tora* was 161,050 bp in length, containing a large single-copy region (LSC) of 90,411 bp, a small single-copy region (SSC) of 18,537 bp, and two inverted repeat (IR) regions of 26,050 bp. The overall GC content is 36.20%, while the correponding values of the LSC, SSC, and IR regions are 64.5%, 69.4%, and 60.2%, respectively. The genome contains 129 complete genes, including 8 rRNAs, 37 tRNAs and 84 protein coding genes. The Neighbour-joining phylogenetic analysis showed that *S. tora* and *S. bicapsularis* clustered together as sisters to other *Senna* species.

## Introduction

*Senna tora* (family: Fabaceae) is ornamental plant belonging to *Cassia* species which is widely distributed in South American and tropical countries. Traditionally, plants belonging to *Cassia* species are believed to possess medicinal values. The wood of *S. tora* can be used to make paper pulp and its fruits are said to be edible (Mak et al. 2013), which is an important medicinal plant for preventing and treating vascular dementia and widely distributed in China, which has persisted largely in an undomesticated state that is highly resistant to different environmental stresses. Vascular dementia refers to a disease caused by cerebrovascular disorders, with dementia as the main clinical phase. Modern pharmacological studies have shown that *S. tora* can significantly increase the number of capillary networks and accelerate blood flow, thereby restoring the function of microcirculation. At the same time, it can also reduce plasma lactic acid content and improve metabolic disorders caused by cell hypoxia. It plays an important role in preventing and treating vascular dementia. Studies have shown that *S. tora* can reduce the content of lipid peroxide and erythrocyte sorbitol, increase the level of superoxide dismutase, reduce the oxidative stress reaction, and has an antioxidant effect. Studies have also found that *S. tora* can improve the role of vascular dementia. The mechanism may be: the antioxidant effect of drugs, by restoring the structure and function of nerve cells, inhibiting lipid peroxidation, improving blood rheology, and protecting against ischemia. Damaged neurons improve the pathological response of vascular dementia. *S. tora* has high ecological and economic value with high levels of intraspecific genetic diversity. *S. tora* has wide geographic distribution, high intraspecific polymorphism, adaptability to different environments, combined with a relatively small genome size. Consequently, *S. tora* represents an excellent model for understanding how different evolutionary forces have sculpted the variation patterns in the genome during the process of population differentiation and ecological speciation (Neale and Antoine 2011). Moreover, we can develop conservation strategies easily when we understand the genetic information of *S. tora*. In the present research, we constructed the whole chloroplast genome of *S. tora* and understood many genome varition information about the species, which will provide beneficial help for population genetics studies of *S. tora.*

A single individual of *S. tora* was used as a sampling object from the Chongqing (105°17 ′E;28°10′N) city. Fresh leaves of the individual were collected and flash-frozen in liquid nitrogen and then stored in a refrigerator (−80 °C) until DNA extraction. The voucher specimen (JMZ001) was laid in the Herbarium of Northwest University and the extracted DNA was stored in the −80 °C refrigerator of the Northwest University. We extracted total genomic DNA from 25 mg silica-gel-dried leaf using a modified CTAB method (Doyle 1987). The Illumina HiSeq 2000 platform (Illumina,San Diego, CA) was used to perform the genome sequence. We used the software MITObim 1.8 (Hahn et al. 2013) and metaSPAdes (Nurk et al. 2017) to assemble chloroplast genomes. We used *S. occidentalis* (GenBank: NC_038222) as a reference genome. We annotated the chloroplast genome with the software DOGMA (Wyman et al. 2004), and then corrected the results using Geneious 8.0.2 (Campos et al. 2016) and Sequin 15.50 (http://www.ncbi.nlm.nih.gov/Sequin/).

The complete chloroplast genome sequence of *Senna tora* (GenBank number: MN863598) was characterized from Illumina pair-end sequencing. The chloroplast genome of *S. tora* was 161,050 bp in length, containing a large single-copy region (LSC) of 90,411 bp, a small single-copy region (SSC) of 18,537 bp, and two inverted repeat (IR) regions of 26,050 bp. The overall GC content is 36.20%, while the correponding values of the LSC, SSC, and IR regions are 64.5%, 69.4%, and 60.2%, respectively. The genome contains 129 complete genes, including 8 rRNAs, 37 tRNAs and 84 protein coding genes.

**Figure 1.**
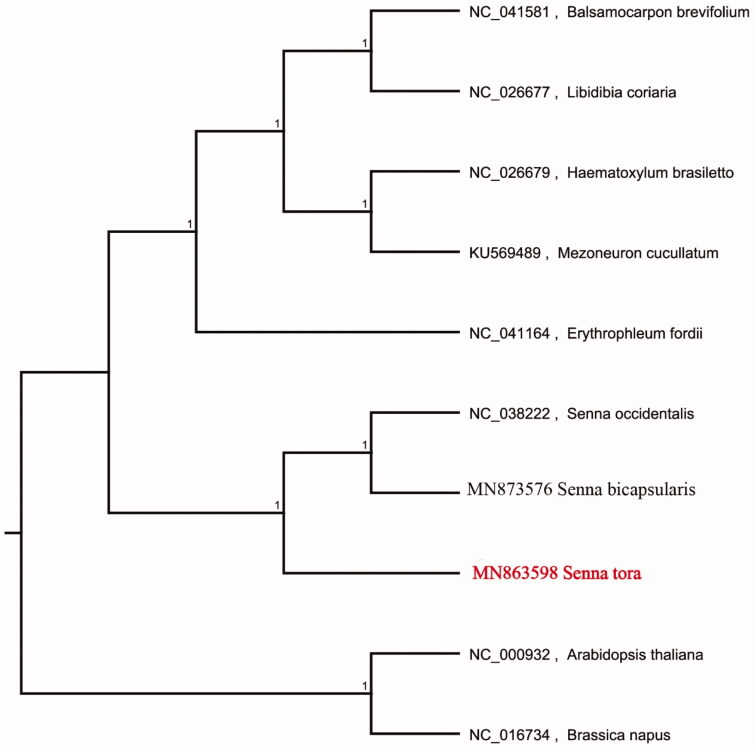
Neighbour-joining (NJ) analysis of *S. tora* and other related species based on the complete chloroplast genome sequence. Genbank accession numbers included in the Figure 1.

We used the complete chloroplast genomes sequence of *S. tora* and 9 other related species and *Brassica napus* and *Arabidopsis thaliana* as outgroup to construct phylogenetic tree. The 10 chloroplast genome sequences were aligned with MAFFT (Katoh and Standley 2013), and then the Neighbour-joining tree was constructed by MEGA 7.0 (Kumar et al. 2016). The results confirmed that *S. tora* was clustered with *S. bicapsularis* (Figure 1).

## Data Availability

The GenBank accession number for the cp genome sequence of *S. tora* is MN863598 and the DOI is https://www.ncbi.nlm.nih.gov.
